# Allelic haplotype combinations at the *MS-P1* region, including P-class pentatricopeptide repeat family genes, influence wide phenotypic variation in pollen grain number through a cytoplasmic male sterility model in citrus

**DOI:** 10.3389/fpls.2023.1163358

**Published:** 2023-06-05

**Authors:** Shingo Goto, Hiroshi Fujii, Hiroko Hamada, Satoshi Ohta, Tomoko Endo, Tokurou Shimizu, Keisuke Nonaka, Takehiko Shimada

**Affiliations:** Citrus Breeding and Production Group, Division of Citrus Research, Institute of Fruit Tree and Tea Science, National Agriculture and Food Research Organization (NARO), Shizuoka, Japan

**Keywords:** CMS, restorer-of-fertility, diplotype, QTL, seedless, marker-assisted selection, PPR

## Abstract

In citrus breeding programs, male sterility is an important trait for developing seedless varieties. Sterility associated with the male sterile cytoplasm of Kishu mandarin (Kishu-cytoplasm) has been proposed to fit the cytoplasmic male sterility (CMS) model. However, it remains undetermined whether CMS in citrus is controlled by interactions between sterile cytoplasm and nuclear restorer-of-fertility (*Rf*) genes. Accordingly, mechanisms underlying the control of the wide phenotypic variation in pollen number for breeding germplasm should be elucidated. This study aimed to identify complete linkage DNA markers responsible for male sterility at the *MS-P1* region based on fine mapping. Two P-class pentatricopeptide repeat (PPR) family genes were identified as candidates for *Rf* based on predicted mitochondrial localization and higher expression in a male fertile variety/selected strain than in a male sterile variety. Eleven haplotypes (HT1–HT11) at the *MS-P1* region were defined based on genotyping of DNA markers. Association analysis of diplotypes at the *MS-P1* region and the number of pollen grains per anther (NPG) in breeding germplasms harboring Kishu-cytoplasm revealed that the diplotypes in this region influenced NPG. Among these haplotypes, HT1 is a non-functional restorer-of-fertility (*rf*) haplotype; HT2, a less-functional *Rf*; HT3–HT5 are semi-functional *Rfs*; and HT6 and HT7 are functional *Rfs*. However, the rare haplotypes HT8–HT11 could not be characterized. Therefore, P-class PPR family genes in the *MS-P1* region may constitute the nuclear *Rf* genes within the CMS model, and a combination of the seven haplotypes could contribute to phenotypic variation in the NPG of breeding germplasms. These findings reveal the genomic mechanisms of CMS in citrus and will contribute to seedless citrus breeding programs by selecting candidate seedless seedlings using the DNA markers at the *MS-P1* region.

## Introduction

Seedlessness is an important trait in citrus because of consumer preference for ease and convenience of eating (Goldenberg et al., 2018). Producing seedless fruits fundamentally requires parthenocarpy and is established *via* a combination of male sterility, female sterility, and self-incompatibility (Vardi et al., 2008; Yamamoto, 2014), whereas male sterility alone effectively reduces seed number in fruits (Yamamoto et al., 1995). Specifically, the male sterility of Satsuma mandarin (*Citrus unshiu* Marcov.) has been frequently used in Japanese citrus breeding programs to develop seedless varieties (Yamamoto, 2014). Both male-sterile and fertile seedlings appear in F_1_ populations with cytoplasm derived from Satsuma mandarin, while all male-fertile seedlings can be obtained from another cytoplasm in a reciprocal cross study; therefore, male sterility is derived from the combined action of cytoplasm derived from Satsuma mandarin and nuclear genes (Yamamoto et al., 1997). Indeed, several studies have supported this phenomenon in male sterility (Yamamoto et al., 1992; Nakano et al., 2001; Dewi et al., 2013; Goto et al., 2016). Additionally, studies have suggested that male sterility could be controlled by the interaction between mitochondria and nuclear genes, as evidenced by male sterility exhibited in cybrid citrus developed with cytoplasm derived from Satsuma mandarin and male-fertile pummelo, Hirado buntan (*C. grandis* [L.] Osbeck) (Zheng et al., 2012; Zheng et al., 2014). These studies have shown that the cytoplasm derived from Satsuma mandarin is an essential genetic factor for the expression of male sterility and is inherited from the seed parent of Kishu mandarin (*C. kinokuni* hort. ex Tanaka) (Fujii et al., 2016; Shimizu et al., 2016). Therefore, the cytoplasm derived from Kishu mandarin (Kishu-cytoplasm) should induce male sterility, suggesting that male sterility in citrus could be categorized as cytoplasmic male sterility (CMS).

Recent studies on CMS across various crops have made remarkable advances (Chen and Liu, 2014). Male sterility is caused by CMS-associated genes in the mitochondrial genome of sterile cytoplasm. Namely, sterility is suppressed by a functional restorer-of-fertility nuclear gene (*Rf*). The male sterile phenotype is a result of interaction between the CMS-associated gene and a non-functional restorer-of-fertility nuclear gene (*rf*) (Chen and Liu, 2014). To elucidate the molecular function of *Rf*, it has been identified in various crops, with maize (*Zea mays*) *Rf2* being the first (Cui et al., 1996). Among the *Rfs* identified, most belong to the pentatricopeptide repeat (PPR) family (Gaborieau et al., 2016). These PPR proteins have a mitochondrial targeting sequence and 11−18 PPR motifs (Dahan and Mireau, 2013), with each motif comprising a 35 amino acid-domain repeated ≤ 30 times in tandem. The PPR family is classified into two subfamilies: P and PLS, where in the P-class subfamily is characterized by tandem arrayed 35 amino acid PPR motifs, and the PLS-class subfamily contains P, L (35 or 36 amino acid variant of PPR motifs), and S (31 amino acid variant) motifs in tandem arrays of characteristic triplets (Lurin et al., 2004). In particular, P-class PPR are identified as *Rfs* for most crops investigated (Dahan and Mireau, 2013). Three nuclear genes that encode PPR proteins and contribute to the cytonuclear interactions in citrus were identified through genome-wide association analysis (Wang et al., 2022); however, there is a lack of molecular evidence to demonstrate that male sterility in the Kishu-cytoplasm fits the CMS genetic model. Further, it is also unclear how the wide phenotypic variations of male sterility and fertility are controlled through the CMS genetic model in citrus breeding germplasms.

Male sterility and fertility segregate in the F_1_ populations of a cross between different varieties and selected strains harboring the Kishu-cytoplasm (Yamamoto et al., 1992; Yamamoto et al., 1997; Nakano et al., 2001; Dewi et al., 2013; Goto et al., 2016). Specifically, the genomes in Japanese varieties and selected strains were constituted by genomic fragments (i.e., haplotype blocks) that are derived from limited founders (Imai et al., 2017). In addition, the traits in each variety/selected strain are influenced by a combination of haplotype blocks (Fujii et al., 2021). These reports raise the possibility that this combination influences the degree of male sterility and fertility of these varieties and selected strains. In a previous study, we have shown that reduced number of pollen grains per anther (NPG) is the primary cause of male sterility in citrus (Goto et al., 2016) and have identified a major quantitative trait locus (QTL) associated with the reduced NPG (*MS-P1*). This reduced NPG is linked to the haplotype block derived from kunenbo in the *MS-P1* locus (Goto et al., 2018). Therefore, the present study aimed to elucidate whether *Rf* are located in the *MS-P1* locus and to determine the influence of a combination of haplotype blocks (including *Rf*) on the phenotypic variations pertaining to male sterility and fertility in individuals with Kishu-cytoplasm.

To elucidate the molecular mechanism underlying male sterility in Kishu-cytoplasm and assess if it fits into the CMS genetic model, the present study aimed to: (1) identify the *MS-P1* region within the locus using fine mapping, (2) identify candidates for *Rf* (*Rf-MS-P1*) through bioinformatic and transcriptional analysis, (3) define the number of allelic haplotypes at the *MS-P1* region among the founders; and (4) reveal the association between NPG and the combination of allelic haplotypes (diplotype) at the *MS-P1* region. Hybrid varieties, selected strains, and individuals in F_1_ populations (breeding germplasms) harboring the Kishu-cytoplasm as well as the various diplotypes in each individual were evaluated. Two mitochondrial-targeted PPR family genes at the *MS-P1* region showed significant transcriptional differences between sterile and fertile varieties during flower development, indicating that they played an important *Rf* role in the CMS model. Assessing the combination allelic haplotypes, including the two PPR family genes, would enhance our understanding of the wide phenotypic variations observed in citrus male sterility and fertility associated with a sterile cytoplasm.

## Materials and methods

### Plant materials and evaluation of male sterility

The three F_1_ populations, Okitsu No. 46 × ‘Kara’ (O46-K), ‘Sweet spring’ × Okistu No. 56 (SS-O56) and ‘Harehime’ × Okistu No. 63 (H-O63), and the varieties/selected strains used in this study were maintained in the Division of Citrus Research, Institute of Fruit Tree and Tea Science, NARO (Shizuoka, Japan) (**Tables S1**, **S2**). The individuals of the F_1_ populations were grafted onto trifoliate orange rootstocks as a single replicate in April 2012 and 2013 (**Table S1**), while the NPG of individuals in the O46-K population had been previously evaluated in 2015 (**Table S1**) (Goto et al., 2016; Goto et al., 2018). The NPG in the varieties/selected strains in 2017 and 2018, O46-K in 2016, SS-O56 in 2015 and 2016, and H-O63 in 2015 and 2016 was evaluated. The protocols used for NPG evaluations are available at protocols.io (dx.doi.org/10.17504/protocols.io.q78dzrw).

### Genomic DNA extraction, design of SSR markers, and genotyping analysis

Genomic DNA was extracted from fresh leaves of the F_1_ populations and the 85 varieties/selected strains through a modified protocol using cetyltrimethylammonium bromide and a high-salt precipitation solution (1.2 M NaCl, 0.8 M sodium citrate; protocol available at dx.doi.org/10.17504/protocols.io.dm6gpj8jpgzp/v1). Six DNA markers (Marker No. 1, 9, 12, 13, 15, 19) in the *MS-P1* locus were used in a previous report (**Table S3**) (Ollitrault et al., 2010; Shimizu et al., 2016), while 13 new SSR markers (Marker No. 2–8, 10, 11, 14, 16–18) in the *MS-P1* locus (**Table S3**) were identified for this study. The Clementine genome sequence between TSRF161 and SSR08B66, which corresponds to scaffold 8, 5,153,769−19,761,190 bp in the physical map of *C. clementina* genome v1.0 (JGI) (Goto et al., 2018), was obtained from Phytozome (https://phytozome-next.jgi.doe.gov/info/Cclementina_v1_0) (Wu et al., 2014). The corresponding scaffolds of Satsuma mandarin from MiDB (https://mikan.dna.affrc.go.jp/) were identified using BLAST (https://mikan.dna.affrc.go.jp/blast/) (Kawahara et al., 2020). The identified scaffolds were screened for dimeric and trimeric SSR sequences with ≥ 7 repeats using the Simple Sequence Repeat Identification Tool (https://archive.gramene.org/db/markers/ssrtool) (Temnykh et al., 2001). The primers for the 13 markers identified based on the detected SSRs were designed using Primer3 v.4.1.0 (https://bioinfo.ut.ee/primer3/) (**Table S3**) (Kõressaar et al., 2018). Dimeric SSRs were preferred for developing markers that can detect regions with high polymorphism, and the multicolored post-labeling method was used for genotyping analysis (Shimizu and Yano, 2011), as described previously (Shimizu et al., 2016).

### Fine mapping of the *MS-P1* region responsible for male sterility using three F_1_ populations

Genotyping was performed with 19 markers (Marker No. 1–19 in **Table S3**) for 34 individuals in O46-K, 31 individuals in SS-O56, and 50 individuals in H-O63 (**Table S1**). Subsequently, recombinant individuals within the *MS-P1* locus were identified based on the genotype segregation pattern. A graphical genotype of the *MS-P1* locus was constructed for the physical map of *C. clementina* genome v1.0 between 5,153,708 and 19,761,190 bp in scaffold 8. The male sterile genotypes within the *MS-P1* locus in recombinant individuals were identified through comparisons with the genotype of male sterile varieties/selected strains (**Figure S1**), whereas the male sterile phenotypes of the recombinant individuals were evaluated through comparison of the anthers from the male sterile and fertile varieties/selected strains (**Figure S1**). The *MS-P1* region was fine mapped considering the associations between the male sterile phenotypes and genotypes in the *MS-P1* locus of recombinant individuals.

### Bioinformatic analysis

The gene locus, annotations, and protein sequences at the *MS-P1* region of *C. clementina* genome v1.0 (JGI) were obtained from Phytozome (https://phytozome-next.jgi.doe.gov/info/Cclementina_v1_0) (Wu et al., 2014). The DNA sequences of maize (*Zea mays*) *Rf2* (U43082), rice (*Oryza sativa*) *Rf2* (AB583697), rice *Rf17* (Os04g0475900), and sugar beet (*Beta vulgaris*) *Rf1* (AB646135), all of which belong to the non-PPR family of *Rf*, were used as the query to perform BLAST analysis against the sequences in the Mikan Genome DB under default settings. The protein sequences of the PPR family at the *MS-P1* region were analyzed using TargetP v.2.0 (https://services.healthtech.dtu.dk/service.php?TargetP-2.0) (Almagro Armenteros et al., 2019), Predotar v.1.04 (https://urgi.versailles.inra.fr/predotar/) (Small et al., 2004), and MitoProt II (https://ihg.helmholtz-muenchen.de/ihg/mitoprot.html) (Claros and Vincens, 1996) to predict their subcellular localization and targeting sequence. To classify the PPR subfamily, PPR protein sequences were analyzed using PPRfinder v.507b0fb (https://ppr.plantenergy.uwa.edu.au/) (Gutmann et al., 2020). Protein sequence alignments were performed using MAFFT v.7 (https://mafft.cbrc.jp/alignment/server/) (Katoh et al., 2019), while PPR motifs were identified using ScanProsite release 20.0 (https://prosite.expasy.org/scanprosite/), and phylogenetic trees were constructed using the neighbor-joining method of MEGA v.10.0.5 (https://www.megasoftware.net) with protein sequences of rice (Oryza sativa) Rf1a (DQ311053), rice Rf1b (DQ311054), rice Rf4 (KJ680249), Sorghum (*Sorghum bicolor*) Rf1 (Klein et al., 2005), Chinese cabbage (*Brassica napus*) Rfp1 (KX671967), Petunia (*Petunia hybrida*) Rf-PPR592 (AY10271), Ciclev10030242m, and Ciclev10030361m. Bootstrap values were calculated through a 1,000-permutation test.

### RNA-seq analysis

Bulked stamen from a sterile selected strain (KyOw14) and fertile variety (‘Shiranuhi’) were collected seven days before flowering (DBF) and 1 DBF in a field from the Division of Citrus Research, Institute of Fruit Tree and Tea Science, NARO (Shizuoka, Japan), as two biological replicates. Total RNA was isolated using an RNeasy Plant Mini Kit (Qiagen, Hilden, Germany), while the RNA-seq analysis was carried out by Hokkaido System Science Co., Ltd. (Sapporo, Japan). RNA-seq libraries were generated from the total RNA using the NEBNext Ultra RNA Library Prep Kit for Illumina (New England BioLabs, Inc., Ipswich, MA). The 150 bp paired-end sequencing of RNA-seq libraries was performed using the NovaSeq 6000 system (Illumina, San Diego, CA), and the sequenced reads were trimmed using fastp v.0.22.0 and mapped to *C. clementina* genome v1.0 (JGI) (https://phytozome-next.jgi.doe.gov/info/Cclementina_v1_0) using HISAT2 v.2.1.0. Transcripts per million (TPM) values were obtained to measure gene expression using StringTie v.2.1.1. TPM values were imported to Subio Platform v.1.24.5853 (Subio Inc., Aichi, Japan) for normalization. Statistical analysis of the comparisons between ‘Shiranuhi’ and KyOw14 was carried out using the “compare 2 groups” tool of the Subio platform. All RNA-seq data was deposited in the DDBJ Sequence Read Archive under accession number DRA015326.

### Gene expression analysis by quantitative RT-PCR

To confirm the reproductivity of the transcriptome analyses concerning the PPR genes localized in the mitochondria, three replicates of the total RNA from the stamen of KyOw14 and ‘Shiranuhi’ at 7 DBF and 1 DBF were used for quantitative RT-PCR and semi-quantitative RT-PCR. Reverse-transcription was carried out using QuantiTect Reverse Transcription Kit (Qiagen, Hilden, Germany). Quantitative PCR was performed QuantStudio3 Real Time PCR System (Thermo Fisher Scientific, Waltham, USA) using Power SYBR Green PCR Master Mix (Thermo Fisher Scientific) under 10 min at 95°C, followed by 40 cycles of 15 s at 95°C and 60 s at 60°C. The primers for the gene expression of Ciclev10030242m (Fd: GATAAGAGAAATGTAATGCCAGACG, Rv: CCTTCACACGTTGTAATAAGAATGG), Ciclev10030361m (Fd: AGAGAAATGTAATGCCAGACGC, Rv: CAGCTCATTGACTTACTTGTGTCTA), and Ciclev10029947m (Fd: ACCCAATTGTGTCATATTTACTACGC, Rv: AATCAACATTCAACTCACCCACTTAC) were designed at a specific region or 3’UTR referred by Phytozome (https://phytozome-next.jgi.doe.gov/info/Cclementina_v1_0) (Wu et al., 2014). The expression of these genes were normalized by that of *eEF1a* (Fd: TCAAGGATCTCAAGCGTGGTT, Rv: CTTCCCTGGCCGGATCAT) (Shimada et al., 2018). Specific amplification in each PCR reaction was confirmed by melting curve analysis and agarose gel electrophoresis.

### Determination of source of cytoplasm

The sources of cytoplasm in the 85 varieties/selected strains were determined referring to the pedigree chart (Imai et al., 2017).

### Haplotyping and diplotyping of 85 varieties/selected strains and F_1_ populations at the *MS-P1* region

Both 00918-2 and 00918-3 were designed on the scaffolds of C_unshiu_00918, as described above (Marker No. 20 and 21 in **Table S3**). Haplotypes were defined based on segregation of the genotyped alleles at 00918-1, 00918-2, and 00918-3 in the three F_1_ populations (O46-K, SS-O56, and H-O63) (**Table S1**), before being classified into seven groups (HT1–HT7). HT3, derived from sweet orange, and HT6, derived from Dancy tangerine, Iyo, Ponkan, or Willowleaf mandarin, were differentiated based on the pedigree chart (Imai et al., 2017), although HT3 and HT6 are indistinguishable by 00918-1, 00918-2 and 00918-3. Additional haplotypes were defined and classified into four groups (HT8–HT11) based on the pedigree chart (Imai et al., 2017) of genotyped alleles, with 00918-1, 00918-2, and 00918-3 in 85 varieties/selected strains (**Table S2**). Finally, the diplotype (combination of haplotypes) in the 85 varieties/selected strains and F_1_ population individuals were determined using the 11 haplotypes and pedigree chart (Imai et al., 2017).

### Statistical associations analysis

Statistical analyses were performed with EZR v.1.37, which is a graphical user interface for Rcmdr v.2.4-0 (https://www.jichi.ac.jp/saitama-sct/SaitamaHP.files/statmedEN.html) (Kanda, 2013).

## Results

### Fine mapping of the *MS-P1* region and associated predicted genes

**Figure 1:**
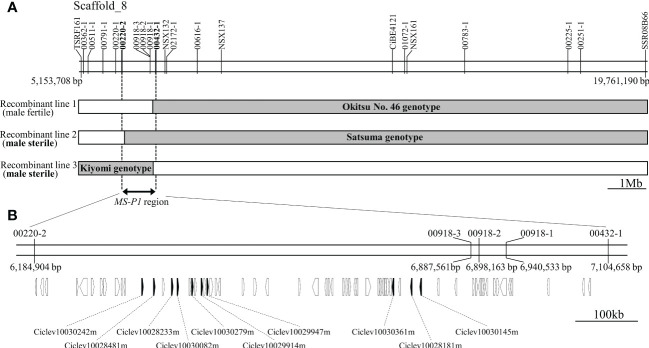
Fine mapping of the *MS-P1* region and the corresponding predicted genes. **(A)** Illustration of the genotypes for the three recombinant individuals with 20 markers. Markers and positions are indicated on the physical map of *C clementina* genome v1.0 (JGI). Recombinant line 1, Recombinant line 2, and Recombinant line 3 were identified from Okitsu No. 46 × Kara, ‘Sweet spring’ × Okistu No. 56, and ‘Harehime’ × Okistu No. 63, respectively. Phenotype (male sterile or fertile) for each individual is shown in brackets. Gray bars indicate male sterile genotype, whereas names in the gray bars show varieties/selected strains with the same genotype as that of each sterile genotype. The *MS-P1* region is indicated using a double-headed arrow. **(B)** Predicted genes at the *MS-P1* region. The physical position of each marker is based on *C clementina* genome v1.0. Arrowhead directions indicate the direction of transcription. Genes annotated as a pentatricopeptide repeat (PPR) family are shown as black arrowheads, with accession number included.

Previously, we identified an *MS-P1* locus between 33.5cM (TSRF161) and 69.1cM (SSR08B66) on linkage group 8 (LG8) in the corresponding linkage map of Okitsu No. 46 × Okitsu No. 56 (Goto et al., 2018). For identifying individual recombinations within the *MS-P1* locus, genotyping was carried out for 19 DNA markers between TSRF161 and SSR08B66 (Marker No. 1−19; **Table S3**) in individuals of three F_1_ populations derived from SS-O56, O46-K, and H-O63 (**Table S1**). The following three recombinant individuals were identified: Recombinant line 1, male fertile; Recombinant line 2, male sterile; and Recombinant line 3, male sterile (**Figure 1A**). Several DNA marker genotypes of these recombinant lines indicated the presence of the same genotypes as that in Okitsu No. 46, Satsuma mandarin, and ‘Kiyomi’ (**Figure 1A**; **Table S2**). Notably, there were no pollen grains on the anthers of these varieties/selected strains, indicative of male sterility (**Figure S1**); therefore, the corresponding genotypes were considered male sterile. Association analysis between the male sterile phenotype and genotypes in the recombinant lines enabled isolation of the *MS-P1* region within 00220-2 and 00432-1, corresponding to a 920kb genomic region from 6,184,904 to 7,104,658 bp of scaffold 8 of *C. clementina* genome v1.0 (JGI) (**Figure 1A**).

### Cluster of PPR family genes on newly refined *MS-P1* region

Based on the annotation information for *C. clementina* genome v1.0 (JGI), 66 functional genes, including 10 PPR family genes, were predicted on the 920 kb genomic region, corresponding to the newly refined *MS-P1* region (**Figure 1B**). The PPR family genes were highly redundant in this genomic region, and a tandem repeat-like cluster was observed (**Figure 1B**; **Table 1**). Genes homologous to maize *Rf2* (U43082), rice *Rf2* (AB583697), rice *Rf17* (Os04g0475900), or sugar beet *Rf1* (AB646135), which are non-PPR families of *Rf*, were not found among the 66 genes here. Bioinformatic analysis using PPRfinder revealed that all PPR family genes at the *MS-P1* region belonged to the P-class subfamily (**Table 1**). The proteins encoded by three genes in the PPR family, Ciclev10030242m, Ciclev10029947m, and Ciclev10030361m, were predicted to localize in the mitochondria using TargetP 2.0 (Almagro Armenteros et al., 2019), Predotar v.1.04 (Small et al., 2004), or MitoProt II (Claros and Vincens, 1996) (**Table 1**).

**Table 1:** Subfamily and predicted subcellular localization of pentatricopeptide repeat.

Gene locus at Scaffold 8	Strand	Accession No.	PPR subfamily	TargetP 2.0 Prediction §	Predotar 1.04 Prediction §	MitoProt II Prediction *
6,355,458–6,359,023	+	Ciclev10030242m	P-class	None	Possibly mitochondria	Mitochondria
6,373,589–6,374,942	+	Ciclev10028481m	P-class	None	None	None
6,402,840–6,404,443	+	Ciclev10028233m	P-class	None	None	None
6,411,573–6,413,114	+	Ciclev10030082m	P-class	None	None	None
6,434,300–6,435,849	+	Ciclev10030279m	P-class	None	None	None
6,451,978–6,453,577	+	Ciclev10029914m	P-class	None	None	None
6,460,563–6,461,664	+	Ciclev10029947m	P-class	Mitochondria	Mitochondria	Mitochondria
6,757,857–6,759,743	–	Ciclev10030361m	P-class	Mitochondria	Plastid or Mitochondria	None
6,790,357–6,792,194	–	Ciclev10028181m	P-class	None	None	None
6,805,621–6,806,699	–	Ciclev10030145m	P-class	None	None	None

§: “None” indicates no targeting sequence

*: “None” indicates no mitochondrial targeting sequence Proteins at the *MS-P1* region. "+" and "-" indicate forward and reverse strand gene, respectively.

### Transcriptomic analysis of PPR family genes at the *MS-P1* region using RNA-seq

**Figure 2:**
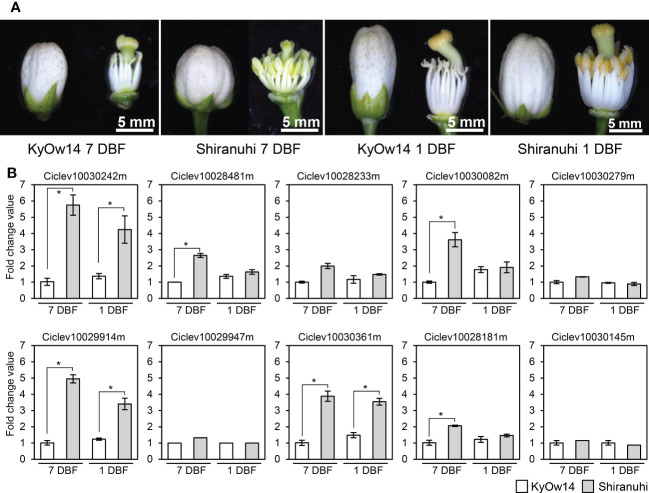
Expression of PPR family genes at the *MS-P1* region evaluated using RNA-seq. **(A)** RNA was extracted from KyOw14 and ‘Shiranuhi’ stamen at 7 and 1 days before flowering (DBF). **(B)** Expression of PPR family genes at the *MS-P1* region in KyOw14 and ‘Shiranuhi’ at 7 and 1 DBF, evaluated using RNA-seq. White and gray bars indicate gene expressions in KyOw14 and ‘Shiranuhi’, respectively. Asterisks indicate significant differences according to the *t*-test (*p* < 0.05).

Assuming that male sterility in the Kishu-cytoplasm fits into the CMS genetic model, the *Rf* corresponding to the Kishu-cytoplasm (*Rf-MS-P1*) should be expressed more in a male fertile variety/selected strain than in a male sterile variety/selected strain. Transcriptomic analyses of KyOw14 (sterile selected strain) and ‘Shiranuhi’ (fertile variety) stamens were performed at 7 and 1 DBF through RNA-seq analysis. RNA extracted from the stamens (two replicates) were used to build libraries for RNA-seq (**Figure 2A**). An average of 26 million reads per sample were obtained (**Table S4**), among which 97% of the reads were passed through quality thresholds, and 95% were mapped to the reference genome (**Table S4**). These data showed that the RNA sequencing quality was highly valid and suitable for comparative analysis. Comparative transcriptomic analysis between the PPR family genes at the *MS-P1* region of KyOw14 and ‘Shiranuhi’ was performed (**Figure 2B**; **Table S5**). The expression of Ciclev10030242m, Ciclev10029914m, and Ciclev10030361m was significantly higher in ‘Shiranuhi’ than in KyOw14 at both 7 and 1 DBF (*p*< 0.05) (**Figure 2B**; **Table S5**). Among them, Ciclev10030242m and Ciclev10030361m were predicted to be localized in the mitochondria (**Table 1**). Expression of Ciclev10029947m, which was also predicted to be localized in the mitochondria (**Table 1**), was hardly detected in either KyOw14 or ‘Shiranuhi’ (**Figure 2B**; **Table S5**). The expression of Ciclev10030242m and Ciclev10030361m was reproduced by quantitative RT-PCR (**Figure S3A**). The expression of Ciclev10029947m was also reproduced by quantitative RT-PCR ((**Figure S3A**) and semi-quantitative RT-PCR (**Figure S3B**). Predicted subcellular localization and transcriptomic analysis indicated Ciclev10030242m and Ciclev10030361m as transcriptionally plausible candidates for *Rf-MS-P1*.

### Protein sequence analysis of *Rf-MS-P1* candidates

**Figure 3:**
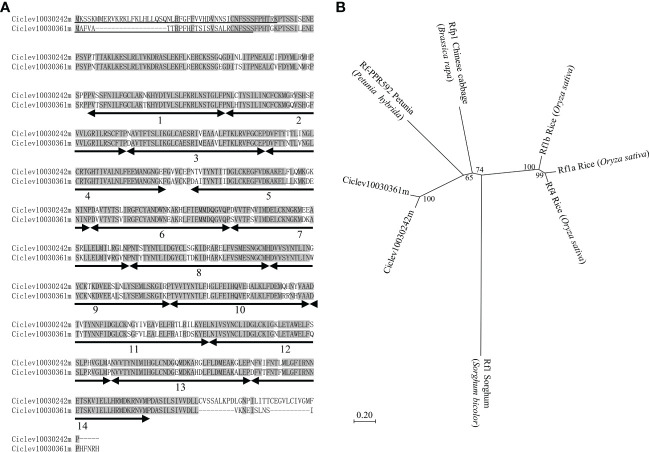
Characterization of *Rf-MS-P1* candidates based on protein sequence. **(A)** Protein sequence alignment of *Rf-MS-P1* candidates. Residues identical in both genes are highlighted in grey. Positions and numbers of pentatricopeptide repeat (PPR) motifs are indicated below the protein sequences using a double-headed arrow, while the predicted mitochondrial targeting sequences are underlined. The targeting sequences of Ciclev10030242m and Ciclev10030361m were predicted using MitoProt II and TargetP 2.0, respectively. **(B)** Phylogenetic analysis of *Rf-MS-P1* candidates in citrus and *Rf* genes in other species. Bootstrap values are shown on branches, and scalebars represent the number of substitutions per site.

The protein sequence analysis showed that Ciclev10030242m and Ciclev10030361m were highly similar proteins, with 86.66% overlap (**Figure 3A**), maintaining 14 PPR motifs with a mitochondrial targeting sequence (**Figure 3A**). Neighbor-joining phylogenetic tree analysis was carried out using protein sequences of Ciclev10030242m, Ciclev10030361m, rice Rf1a (DQ311053), rice Rf1b (DQ311054), rice Rf4 (KJ680249), sorghum Rf1 (Klein et al., 2005), Chinese cabbage Rfp1 (KX671967), and petunia Rf-PPR592 (AY10271), all of which are PPR family Rfs. The result showed that Ciclev10030242m and Ciclev10030361m were clustered together (**Figure 3B**). Notably, the cluster was closer to that of Chinese cabbage Rfp1 and petunia Rf-PPR592 than to that of rice Rfs (**Figure 3B**). Further, sorghum Rf1 was far from the other clusters because it alone was included in the PLS-class subfamily (**Figure 3B**) (Dahan and Mireau, 2013). Therefore, Ciclev10030242m and Ciclev10030361m were potential candidates for *Rf-MS-P1*, as they belonged to the P-class PPR family, had mitochondrial targeting sequences, and were expressed more in ‘Shiranuhi’ (fertile variety) than in KyOw14 (sterile strain). In addition, the existence of Ciclev10030242m and Ciclev10030361m at the *MS-P1* region provides evidence that molecular mechanisms underlying citrus male sterility fit into the general plant CMS model.

### Identification of allelic haplotype at the *MS-P1* region among Japanese breeding germplasm founders

The molecular mechanism of citrus male sterility fits within the general plant CMS model; however, it remains unclear how the wide phenotypic variations of male sterility and fertility observed in Japanese breeding germplasms are controlled. It was hypothesized here that male sterility is influenced by a combination of allelic haplotypes in which *Rf* or *rf* are located; therefore, we defined allelic haplotypes at the *MS-P1* region among the founders of Japanese breeding germplasm. The nearest markers to candidates for *Rf-MS-P1* were narrowed down to 00918-1 through fine mapping of the *MS-P1* region (**Figure 1A**). We focused on the region containing 00918-1 and developed two markers, 00918-2 and 00918-3 in the proximity of 00918-1 using the genome sequence of C_unshiu_00918 in MiDB (**Figure 1**; Marker No. 20 and 21 in **Table S3**). Each allelic haplotype was determined based on the genotype of these three markers. The 85 varieties/selected strains (**Tables S2**; **S6A**) and the three F_1_ populations (SS-O56, O46-K, H-O63) were genotyped (**Tables S1**; **S6B**) with the 00918-1, 00918-2, and 00918-3 markers. The parent-offspring diagnosis did not show discrepancies in the obtained genotypes for any of the varieties/selected strains and the three F_1_ populations except for ‘Willking’ (see legend in **Table S6A**). A total of 11 haplotypes were defined (HT1–HT11) among the 12 founder varieties, with three genotyped alleles at 00918-1, 00918-2, and 00918-3 (**Table 2**). The reduced NPG was linked to the haplotype block derived from kunenbo in the *MS-P1* locus (Goto et al., 2018), which was identified as HT1 in this study (**Table 2**). Okitsu No. 46 harbored HT1 and HT3 derived from kunenbo and sweet orange, respectively (**Table S6A**). Okitsu No. 46 showed reduced NPG (**Figure S1**; **Table S6A**), and individuals with reduced NPG appeared in the progeny of Okitsu No. 46 (Goto et al., 2016; Goto et al., 2018); therefore, HT3 was associated with the reduced NPG and was defined as a haplotype that harbored the “248” allele at 00918-1, the “234” allele at 00918-2, and the “221” allele at 00918-3 derived from sweet orange (**Table 2**). HT6 was defined as a haplotype comprising same alleles as HT3 derived from Dancy tangerine (*C. tangerina* hort. ex Tanaka), Iyo (*C. iyo* hort. ex Tanaka), Ponkan (*C. reticulata* Blanco), or Willowleaf mandarin (*C. deliciosa* Ten.) (**Table 2**). The three alleles comprising each haplotype were completely linked to each other in all varieties/selected strains and individuals in the three F_1_ populations, indicating that they were inherited as a haplotype blocks. The diplotype in the 85 varieties/selected strains and the three F_1_ populations was determined based on the 11 haplotypes (**Tables S6A, B**). HT3 and HT6 were distinguished based on the pedigree chart (Imai et al., 2017); however, several varieties/selected strains were indistinguishable from each other (**Table S6A**)

**Table 2:** Allelic composition and presumed function of haplotypes at the *MS-P*1 region.

Haplotype	Allele of 00918-1*	Allele of 00918-2*	Allele of 00918-3*	Founders from which the haplotype was derived	Appearance frequency	Function as a restorer-of-fertility
HT1	260	238	223	Kunenbo, Hassaku, Hyuganatsu, King, Grapefruit, Murcott	63	Non-functional restorer-of-fertility
HT2	252	197	221	Sweet orange	20	Less-functional restorer-of-fertility
HT3	248	234	221	Sweet orange	9	Semi-functional restorer-of-fertility
HT4	248	214	221	King, Murcott	8	Semi-functional restorer-of-fertility
HT5	256	218	221	Kishu, Kunenbo	13	Semi-functional restorer-of-fertility
HT6	248	234	221	Dancy tangerine, Ponkan, Willowleaf mandarin, Iyo	33	Functional restorer-of-fertility
HT7	248	236	221	Ponkan, Willowleaf mandarin	6	Functional restorer-of-fertility
HT8	272	232	225	Hyuganatsu	3	Undetermined
HT9	248	224	223	Hassaku	2	Undetermined
HT10	254	238	223	Kishu	1	Undetermined
HT11	256	191	221	Iyo	1	Undetermined

*: Number indicates PCR fragment size of each SSR marker

Appearance frequency refers to that of each haplotype at the MS-P1 region in 85 varieties/selected strains. The haplotype of “HT3 or HT6” was excluded from appearance frequency.

### Association between diplotype at the *MS-P1* region and NPG in the breeding germplasms harboring the Kishu-cytoplasm

**Figure 4:**
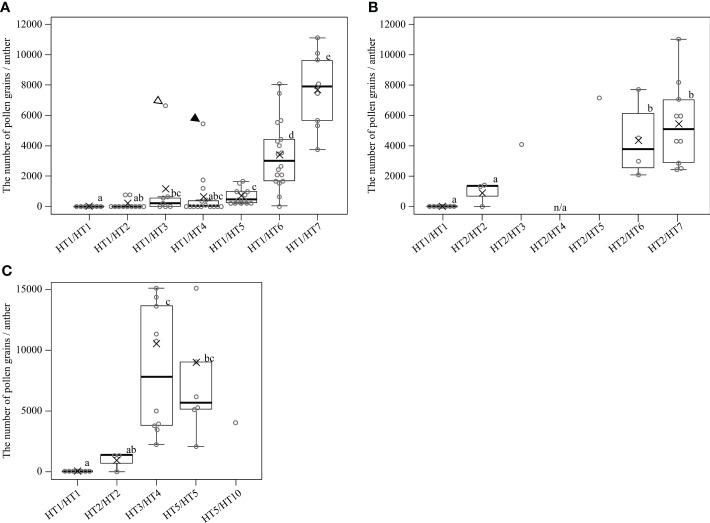
Associations between the number of pollen grains per anther and diplotype at the *MS-P1* region in the individuals of F_1_ populations, and varieties/selected strains with the cytoplasm derived from Kishu mandarin. Diplotype of individuals in the F_1_ populations for SS-O56, O46-K, and H-O63. Varieties/selected strains (breeding germplasms) were determined based on alleles at 00918-1, 00918-2, and 00918-3. Box plots overlaid with dot plots represent the number of pollen grains per anther. Black solid line and cross mark indicate the median and average number of breeding germplasms classified into each diplotype, respectively. Boxes demarcate lower and upper quartiles, whereas the upper and lower adjacent lines show the maximum and minimum values, respectively. Dot plots show the average of each breeding germplasm for 2 years, and dot plots above the maximum line represent outliers. “n/a” indicates “not available” due to alternate bearing. White and black arrowheads indicate ‘Nishinokaori’ and ‘Kara’, respectively. Single data were excluded from statistical analysis. The data showed neither a normal distribution nor homoscedasticity according to a one-sample Kolmogorov–Smirnov test and Bartlett test **(A–C)**, respectively. Statistical analysis was carried out using Mann-Whitney U test and adjusted using Holm’s method. Boxes with the same lower-case letter are not significantly different (*p* > 0.05).

Evaluating the appearance frequency of haplotypes at the *MS-P1* region showed that HT1 was the most frequent haplotype, followed by HT6 and HT2 among the 85 varieties/selected strains (**Table 2**). Japanese breeding programs primarily use Satsuma mandarin and ‘Kiyomi’ as seed parents (**Figure S2**) (Imai et al., 2017), both of which have HT1, which is derived from kunenbo. ‘Kiyomi’ also has HT2, which is derived from sweet orange (**Figure S2**); thus, HT1 or HT2 was present in all varieties used in the present study during 1st and 2nd generation breeding (**Figure S2**). This constitutes one of the main reasons why HT1 and HT2 were frequently observed haplotypes. The diplotypes in the varieties/selected strains with Kishu-cytoplasm predominantly showed combinations of HT1 and HT2 (**Table S6A**); therefore, focus was placed on HT1 and HT2 at the *MS-P1* region, and the association between the NPG and combinations of HT1/HT2 as well as another haplotype in the F_1_ populations of SS-O56, O46-K, and H-O63 and the varieties/selected strains harboring the Kishu-cytoplasm (breeding germplasms) were investigated (**Tables S6A, B**). Subsequently, NPGs in the breeding germplasms were evaluated for two years (**Tables S6A, B**). First, to investigate the association between the NPG and the combination of HT1 and another haplotype, breeding germplasms which have HT1/HT1, HT1/HT2, HT1/HT3, HT1/HT4, HT1/HT5, HT1/HT6, or HT1/HT7 were selected based on the diplotype at the *MS-P1* region (**Figure 4A**). Association analysis revealed that the NPGs in HT1/HT1, HT1/HT2, HT1/HT3, HT1/HT4, and HT1/HT5 were significantly lower than those in HT1/HT6 and HT1/HT7 (*p*< 0.05) (**Figure 4A**). Near zero pollen grains were detected in HT1/HT1 (**Figure 4A**), where the average NPG (7 pollen grains) was lower than that in HT1/HT2 (138), HT1/HT3 (1162), HT1/HT4 (636), and HT1/HT5 (688) (**Figure 4A**). Additionally, the NPG in HT1/HT2 was lower than that in HT1/HT3, HT1/HT4, and HT1/HT5 (**Figure 4A**). The NPG in HT1/HT6 and HT1/HT7 was relatively high, although they harbored HT1. Two varieties, ‘Nishinokaori’ and ‘Kara’, deviated from these results (**Figure 4A**, white and black arrows, respectively; **Table S6A**). Recombination was not observed between 00220-2 and 00432-1, which were flanking markers for 00918-1, -2, and -3 at the *MS-P1* region (**Figure 1**) of ‘Nishinokaori’ and ‘Kara’ (data not shown). Further, to investigate the association between the NPG and the combination of HT2 and another haplotype, the breeding germplasms that have HT2/HT2, HT2/HT3, HT2/HT5, HT2/HT6, or HT2/HT7 were selected (**Figure 4B**). Here, HT1/HT1 was used as the control. Association analysis showed that the average NPG in HT2/HT2 (922) was significantly lower than that in HT2/HT6 (4349) and HT2/HT7 (5466) (*p* < 0.05) (**Figure 4B**). Second, the average NPG in HT2/HT2 was higher than that in HT1/HT1, though this difference was not significant. ‘Asumi’ harbored HT2/HT3, with an average NPG of 4079; whereas Kuchinotsu No. 44 harbored HT2/HT5, and its average NPG was 7216 (**Figure 4B**; **Table S6A**). Third, to investigate the association between NPG and combinations of haplotypes without HT1 and HT2, breeding germplasms that have HT3/HT4, HT5/HT5, and HT5/HT10 were selected, while HT1/HT1 and HT2/HT2 were used as the controls (**Figure 4C**). The average NPG in HT3/HT4 (8383) and HT5/HT5 (7148) was significantly higher than that in HT1/HT1 (*p* < 0.05) (**Figure 4C**); whereas the average NPG in HT3/HT4 and HT5/HT5 was higher than that in HT2/HT2 (**Figure 4C**). Kishu mandarin harbored HT5/HT10, and its average NPG was 4070 (**Figure 4C**; **Figure S6A**). These results reveal that male sterility in Kishu-cytoplasm was influenced by a combination of allelic haplotypes at the *MS-P1* region. In addition, HT1 and HT2 reduced NPG levels, while HT6 and HT7 increased NPG.

## Discussion

This study identified the precise *MS-P1* region (**Figure 1A**). In addition, two candidates for *Rf-MS-P1* at the *MS-P1* region were identified, both of which were P-class PPR family genes predicted to localize to the mitochondria and were expressed more in the fertile variety than in the sterile selected strain (**Figures 1B**, **2B**, **3A**; **Table 1**). A total of 11 haplotypes were identified at the *MS-P1* region and labeled HT1–HT11 (**Table 2**). Further, the NPG was influenced by the diplotype at the *MS-P1* region in varieties/selected strains and F_1_ populations harboring the Kishu-cytoplasm (breeding germplasms) (**Figure 4**).

### Candidates for *Rf-MS-P1*

Proteins encoded by the major *Rfs* contain PPR motifs, localize to the mitochondria, and belong to the P-class subfamily (Lurin et al., 2004; Gaborieau et al., 2016). Ciclev10030242m, Ciclev10029947m, and Ciclev10030361m were annotated to the P-class PPR family genes, and their protein sequences predicted localization to the mitochondria (**Table 1**). Ciclev10030242m and Ciclev10030361m were expressed more in the male fertile variety, ‘Shiranuhi’ (which harbored HT1/HT6), than in the male sterile selected strain, KyOw14 (which harbored HT1/HT1) (**Figure 2B**; **Figure S3A**; **Table S6A**). KyOw14 was obtained from the ‘Kiyomi’ × Satsuma mandarin cross and ‘Shiranuhi’ from ‘Kiyomi’ × Ponkan (**Table S2**). Therefore, KyOw14 and ‘Shiranuhi’ have the same female parent and their male parents, Satsuma mandarin and Ponkan are classified as admixture mandarins based on genomic and phylogenetic analysis (Wu et al., 2018). These show that the genetic background of KyOw14 and ‘Shiranuhi’ are very close. In addition, the diplotype at *MS-P1* in KyOw14 was HT1/HT1, which is homozygous non-functional restorer-of-fertility, and that in ‘Shiranuhi’ was HT1/HT7, which is non-functional restorer-of-fertility/functional restorer-of-fertility (**Table 2**; **Table S6**). These data show that KyOw14 and ‘Shiranuhi’ are the optimal combination for transcriptome comparison in the genes at the *MS-P1* region, suggesting that Ciclev10030242m and Ciclev10030361m are certainly functional in the fertile individual. Taken together, these observations show that Ciclev10030242m and Ciclev10030361m are the most likely potential candidates for *Rf-MS-P1*. Moreover, three nuclear genes that encode PPR proteins contribute to the cytonuclear interactions in citrus at Chr 3, Chr 4, and Chr 7 (Wang et al., 2022). In contrast, the candidates for *Rf-MS-P1* identified in this study were located on Chr 8; therefore, to the best of our knowledge, this study is the first to identify potential candidates for *Rf* in citrus.

### Mechanism of male sterility in Kishu-cytoplasm fits the CMS genetic model

Ciclev10030242m and Ciclev10030361m were identified as candidates for *Rf-MS-P1*. The presence of these genes at the *MS-P1* region provides evidence that male sterility in the Kishu-cytoplasm fits the CMS genetic model. In this model, the presence or absence of expressed male sterility is explained based on the normal cytoplasm, sterile cytoplasm, *rf*, and *Rf* (Chen and Liu, 2014). Male fertility is exhibited in individuals with normal cytoplasm, regardless of the nuclear gene. In addition, male sterility is expressed in individuals with male sterile cytoplasm and homozygous *rf*; therefore, even if individuals have male sterile cytoplasm, male sterility is suppressed, and male fertility is expressed in cases harboring *Rf*. A previous study has shown that heterozygosity for *rf* and semi-functional *Rf* partially restore male sterility, resulting in the expression of partial male sterility; however, homozygous individuals for the semi-functional *Rf* completely restored male sterility (Arakawa et al., 2019). The present study showed that the diplotype at the *MS-P1* region influenced the NPG of breeding germplasms, as indicated by the association analysis (**Figures 4A–C**). The NPG was negligible in those harboring homozygous HT1 at the *MS-P1* region, indicating that these individuals exhibited complete male sterility (**Figure 4A**). Considering the CMS genetic model, this result shows that HT1 was the *rf* haplotype (**Table 2**). In contrast, > 2000 pollen grains per anther were detected in almost all individuals harboring HT1/HT6 and HT1/HT7 (**Figure 4A**), indicative of their exhibited male fertility. Considering HT1 as the *rf* haplotype, this result shows that HT6 and HT7 are the *Rf* haplotypes (**Table 2**). In addition, the average NPG was 138 for individuals harboring HT1/HT2 and 922 for those harboring HT2/HT2 (**Figures 4A, B**), showing that HT2 was the less-functional *Rf* haplotype (**Table 2**). NPG values > 2000 were detected in individuals harboring HT2/HT6 and HT2/HT7, supporting that HT6 and HT7 are *Rf* haplotypes (**Figure 4B**). Furthermore, the NPG range in individuals harboring HT1/HT3, HT1/HT4, and HT1/HT5 (**Figure 4A**) was 636−1162, indicating that they exhibited partial male sterility. Approximately 2000−15,000 NPG were detected in individuals harboring HT2/HT3, HT2/HT5, HT3/HT4, and HT5/HT5 (**Figures 4B, C**). Considering the results of Arakawa et al. (Arakawa et al., 2019), findings from the present study suggest that HT3, HT4, and HT5 are semi-functional *Rf* haplotypes (**Table 2**). Comparatively, the function of restorer-of-fertility in HT8, HT9, HT10, and HT11 could not be determined owing the lack of sufficient data (**Table 2**). The physiological and diplotype analysis together provided enough evidence to support that the molecular mechanism underlying male sterility in the Kishu-cytoplasm fits the CMS genetic model. In addition, these results also show that the NPG in the breeding germplasms were influenced by the combination of allelic haplotypes at the *MS-P1* region.

The results here also suggest that *Rf-MS-P1* exists in a genome region including HT6 or HT7, which are the *Rf* haplotypes (**Table 2**). A region on *C. clementina* genome v1.0 (JGI) corresponding to the *MS-P1* region was derived from Willowleaf mandarin, which harbors HT6 (**Table 2**) (Ollitrault et al., 2012); therefore, the genome region must contain *Rf-MS-P1*. Accordingly, the use of the sequence of *C. clementina* genome v1.0 (JGI) to identify *Rf-MS-P1* candidates was a reasonable strategy.

### Distorted phenotype between NPG and diplotype at *MS-P1* in ‘Nishinokaori’ and ‘Kara’

‘Nishinokaori’ and ‘Kara’ exhibited a discrepancy between the NPG and diplotype at *MS-P1* (**Figure 4A**; **Table S6A**); however, recombinations between markers around the *MS-P1* region were not observed (data not shown). ‘Nishinokaori’ and ‘Kara’ exhibited an increased NPG, although ‘Nishinokaori’ harbored HT1/HT3 and ‘Kara’ harbored HT1/HT4, both of which are partial male sterile diplotypes (**Figure 4A**; **Table S6A**). Further, two independent *Rf* loci corresponding to one CMS-associated gene exist in sorghum, radish, wheat, and rice (Yasumoto et al., 2009; Jordan et al., 2010; Castillo et al., 2014; Tang et al., 2017). Minor QTLs associated with the NPG (*MS-P2* and *MS-P3*) were detected in our previous study (Goto et al., 2018); thus, these loci may distort the NPG in ‘Nishinokaori’ and ‘Kara’.

### Application of findings in a breeding program for seedless citrus

The NPG varied from 1 to 1,800 in the Satsuma mandarin strains from 2017 and 2018 (**Table S6A**). Therefore, they exhibited partial male sterility according to the CMS genetic model; however, the pollen grains were not released from the inside of anthers. Accordingly, it was proposed here that Satsuma mandarin has male sterility contributing to seedless fruits (Goto et al., 2018). Considering these previous finding, along with those of the present study, an NPG of < 1,800 pollen grains per anther was proposed as a criterion of male sterility for seedless citrus breeding. Here, an NPG of < 1,800 was detected in breeding germplasms harboring HT1/HT1, HT1/HT2, HT1/HT3, HT1/HT4, HT1/HT5 (**Figure 4A**), and HT2/HT2 (**Figure 4B**); thus, these breeding germplasms maintain male sterility for seedless citrus breeding. Male sterile individuals can be selected from F_1_ populations through screening for individuals harboring HT1/HT1, HT1/HT2, HT1/HT3, HT1/HT4, HT1/HT5, and HT2/HT2, which are diplotypes genotyped using 00918-1, 00918-2, and 00918-3 at the *MS-P1* region.

HT1 was the most frequent haplotype, followed by HT6 and HT2 among the 85 varieties and selected strains (**Table 2**). This may have been because Japanese breeders primarily used Satsuma mandarin and ‘Kiyomi’ as seed parents (**Figure S2**). Additionally, the selection of seedless lines could have resulted in a higher frequency of HT1 and HT2, as both contribute to seedlessness (**Table 2**). HT6 is an *Rf* haplotype; however, it was the second most frequent haplotype. ‘Encore’, Ponkan, and their progenies, which have the HT6 haplotype, have been frequently used as parents for citrus breeding programs in Japan (Imai et al., 2017), likely indicating why HT6 was the second most frequent haplotype observed here.

In hybridization-based citrus breeding programs, only the screening of large numbers of seedlings can guarantee the identification of new varieties with good quality traits; however, citrus has a long juvenile phase and requires extended periods for evaluating fruit quality. Therefore, the selection of novel variety candidates is both time- and cost-prohibitive (Raveh et al., 2020); although this limitation can be overcome by applying marker-assisted selection at the seedling stage (Roose, 2007). Accordingly, the findings of the present study can aid in marker-assisted selection for seedless citrus breeding through screening male sterile individuals in F_1_ populations.

This study has a limitation that should be addressed in future research. *Rf-MS-P1* candidates were predicted to localize in the mitochondria by only bioinformatic programs. In addition to further fine mapping of *MS-P1* region, cellular localization analysis of Rf-MS-P1candidates could help identify the actual *Rf-MS-P1*. In addition, the results here suggest that *Rf* loci are also present, in addition to *Rf-MS-P1*. Accordingly, identifying all *Rf* loci, and identifying the *Rf* genes at the *Rf* loci will enable a deeper understanding of the CMS mechanism in citrus

## Conclusion

In this study, two potential candidates for *Rf-MS-P1* at the *MS-P1* region were identified. Their protein sequences were annotated to P-class PPR family genes and predicted to be localized to the mitochondria. The two *Rf-MS-P1* candidates were expressed more in the male fertile variety than in a male sterile selected strain. Further, the molecular mechanism underlying male sterility in the Kishu-cytoplasm fit into the CMS genetic model, as observed through the functioning of haplotypes at the *MS-P1* region. This study elucidated a portion of the CMS mechanism in citrus and can contribute to seedless citrus breeding programs. Identifying the actual *Rf* gene through future studies would enable further understanding of the CMS mechanism in citrus.

## Data availability statement

The original contributions presented in the study are publicly available. This data can be found here: DDBJ, accession DRA015326 (https://ddbj.nig.ac.jp/resource/sra-submission/DRA015326).

## Author contributions

SG acquired funding, designed the study, performed experiments, analyzed data, and wrote the original draft. SG, HF, TE, ToS, and TaS designed the study. HH, SO, and KN produced and managed materials. SG and TaS reviewed and edited this manuscript. All authors contributed to the article and approved the submitted version.
