# Corrigendum: Allelic haplotype combinations at the *MS-P1* region, including P-class pentatricopeptide repeat family genes, influence wide phenotypic variation in pollen grain number through a cytoplasmic male sterility model in citrus

**DOI:** 10.3389/fpls.2024.1407558

**Published:** 2024-04-15

**Authors:** Shingo Goto, Hiroshi Fujii, Hiroko Hamada, Satoshi Ohta, Tomoko Endo, Tokurou Shimizu, Keisuke Nonaka, Takehiko Shimada

**Affiliations:** Citrus Breeding and Production Group, Division of Citrus Research, Institute of Fruit Tree and Tea Science, National Agriculture and Food Research Organization (NARO), Shizuoka, Japan

**Keywords:** CMS, Restorer-of-fertility, diplotype, QTL, seedless, marker-assisted selection, PPR

In the published article, there were two errors in **Table 2** as published.

“Kishu” was shown in row “HT5” of column “Founders from which the haplotype was derived” in the published article. However, “Kishu, Kunenbo” is correct.

“Kishu, Kunenbo” was shown in row “HT10” of column “Founders from which the haplotype was derived” in the published article. However, “Kishu” is correct.

The corrected **Table 2** appears below.

**Table 2:** Allelic composition and presumed function of haplotypes at the *MS-P1* region.

Haplotype	Allele of 00918-1*	Allele of 00918-2*	Allele of 00918-3*	Founders from which the haplotype was derived	Appearance frequency	Function as a restorer-of-fertility
HT1	260	238	223	Kunenbo, Hassaku, Hyuganatsu, King, Grapefruit, Murcott	63	Non-functional restorer-of-fertility
HT2	252	197	221	Sweet orange	20	Less-functional restorer-of-fertility
HT3	248	234	221	Sweet orange	9	Semi-functional restorer-of-fertility
HT4	248	214	221	King, Murcott	8	Semi-functional restorer-of-fertility
HT5	256	218	221	Kishu, Kunenbo	13	Semi-functional restorer-of-fertility
HT6	248	234	221	Dancy tangerine, Ponkan, Willowleaf mandarin, Iyo	33	Functional restorer-of-fertility
HT7	248	236	221	Ponkan, Willowleaf mandarin	6	Functional restorer-of-fertility
HT8	272	232	225	Hyuganatsu	3	Undetermined
HT9	248	224	223	Hassaku	2	Undetermined
HT10	254	238	223	Kishu	1	Undetermined
HT11	256	191	221	Iyo	1	Undetermined

The authors apologize for this error and state that this does not change the scientific conclusions of the article in any way. The original article has been updated.

