# Supplementary material for: Allelic haplotype combinations at the MS-P1 region, including P-class pentatricopeptide repeat family genes, influence wide phenotypic variation in pollen grain number through a cytoplasmic male sterility model in citrus
Source: Front Plant Sci. 2023 Jun 5;14:1163358. doi: 10.3389/fpls.2023.1163358 (PMC10278581; doi:10.3389/fpls.2023.1163358)
Supplement: Supplementary file 1 [file Presentation_1.pptx]

## Slide 1
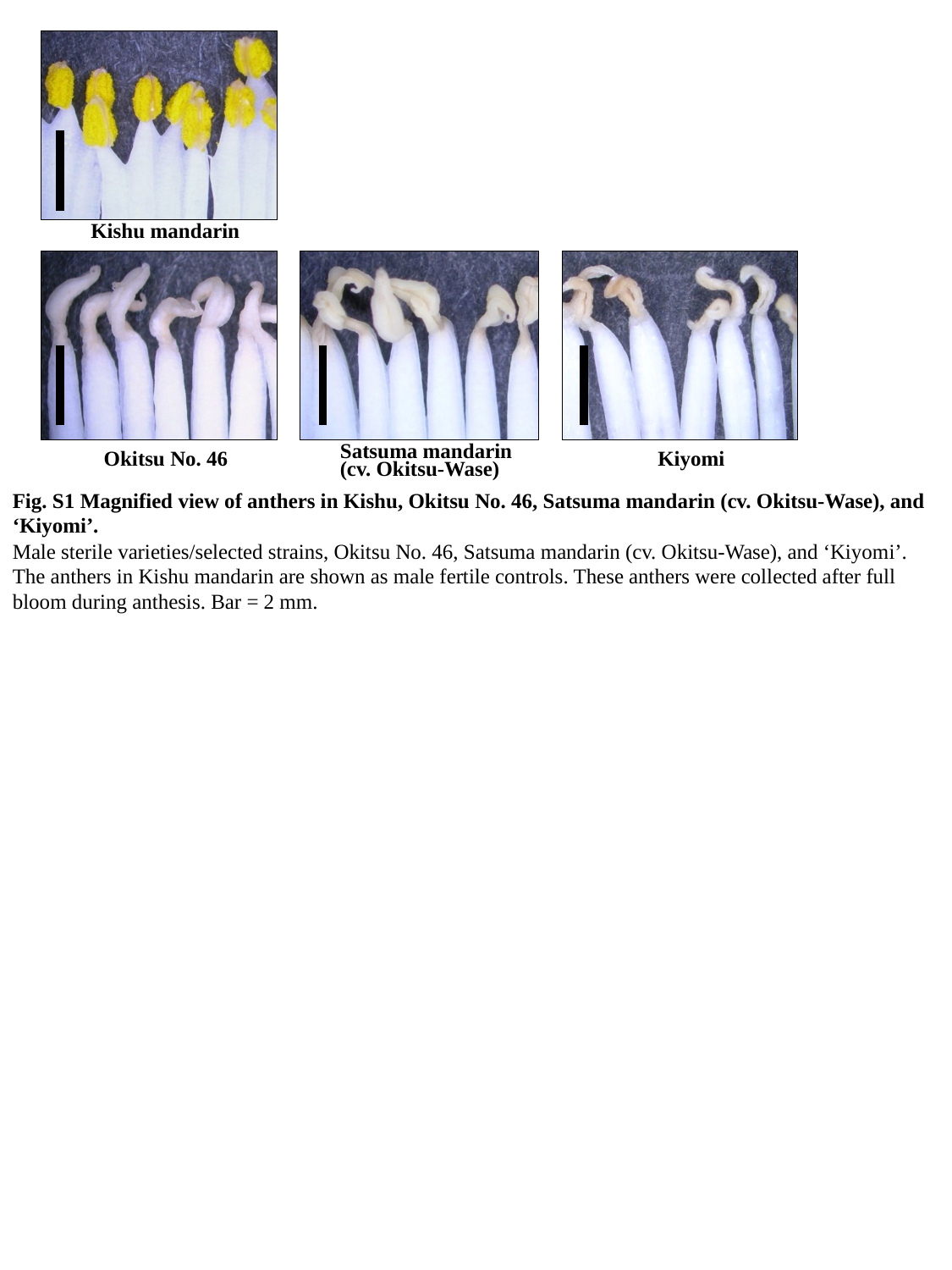

Kishu mandarin
Okitsu No. 46
Satsuma mandarin
(cv. Okitsu-Wase)
Kiyomi
Fig. S1 Magnified view of anthers in Kishu, Okitsu No. 46, Satsuma mandarin (cv. Okitsu-Wase), and ‘Kiyomi’.
Male sterile varieties/selected strains, Okitsu No. 46, Satsuma mandarin (cv. Okitsu-Wase), and ‘Kiyomi’. The anthers in Kishu mandarin are shown as male fertile controls. These anthers were collected after full bloom during anthesis. Bar = 2 mm.
