# Supplementary material for: Allelic haplotype combinations at the MS-P1 region, including P-class pentatricopeptide repeat family genes, influence wide phenotypic variation in pollen grain number through a cytoplasmic male sterility model in citrus
Source: Front Plant Sci. 2023 Jun 5;14:1163358. doi: 10.3389/fpls.2023.1163358 (PMC10278581; doi:10.3389/fpls.2023.1163358)
Supplement: Supplementary file 2 [file Presentation_2.pptx]

## Slide 1
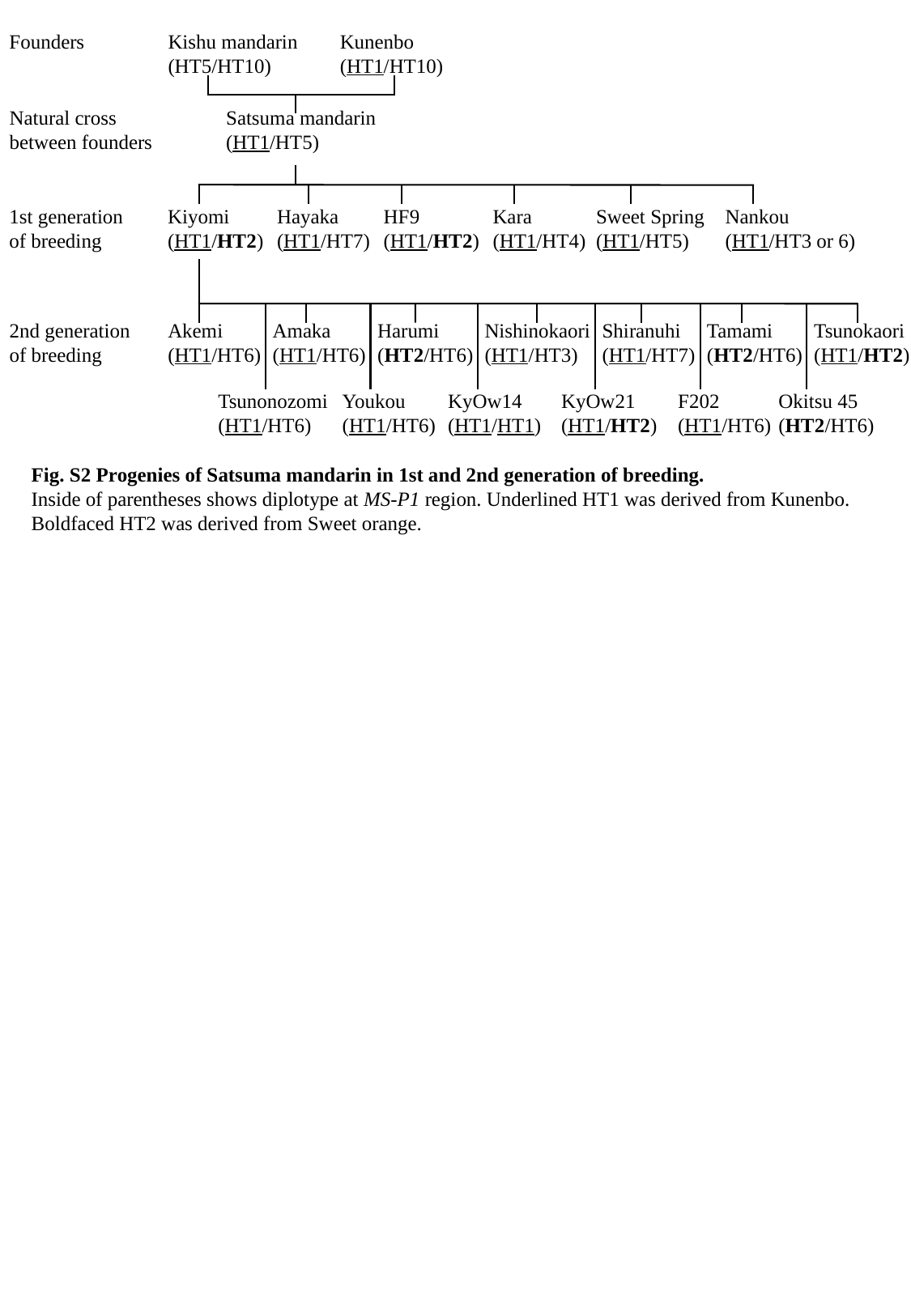

Founders
Kishu mandarin
(HT5/HT10)
Kunenbo
(HT1/HT10)
Natural cross between founders
Satsuma mandarin
(HT1/HT5)
1st generation of breeding
Kiyomi
(HT1/HT2)
Hayaka
(HT1/HT7)
HF9
(HT1/HT2)
Kara
(HT1/HT4)
Sweet Spring
(HT1/HT5)
Nankou
(HT1/HT3 or 6)
2nd generation of breeding
Akemi
(HT1/HT6)
Amaka
(HT1/HT6)
Harumi
(HT2/HT6)
Nishinokaori
(HT1/HT3)
Shiranuhi
(HT1/HT7)
Tamami
(HT2/HT6)
Tsunokaori
(HT1/HT2)
Tsunonozomi
(HT1/HT6)
Youkou
(HT1/HT6)
KyOw14
(HT1/HT1)
KyOw21
(HT1/HT2)
F202
(HT1/HT6)
Okitsu 45
(HT2/HT6)
Fig. S2 Progenies of Satsuma mandarin in 1st and 2nd generation of breeding.
Inside of parentheses shows diplotype at MS-P1 region. Underlined HT1 was derived from Kunenbo. Boldfaced HT2 was derived from Sweet orange.
