# Supplementary material for: Allelic haplotype combinations at the MS-P1 region, including P-class pentatricopeptide repeat family genes, influence wide phenotypic variation in pollen grain number through a cytoplasmic male sterility model in citrus
Source: Front Plant Sci. 2023 Jun 5;14:1163358. doi: 10.3389/fpls.2023.1163358 (PMC10278581; doi:10.3389/fpls.2023.1163358)
Supplement: Supplementary file 3 [file Presentation_3.pptx]

## Slide 1
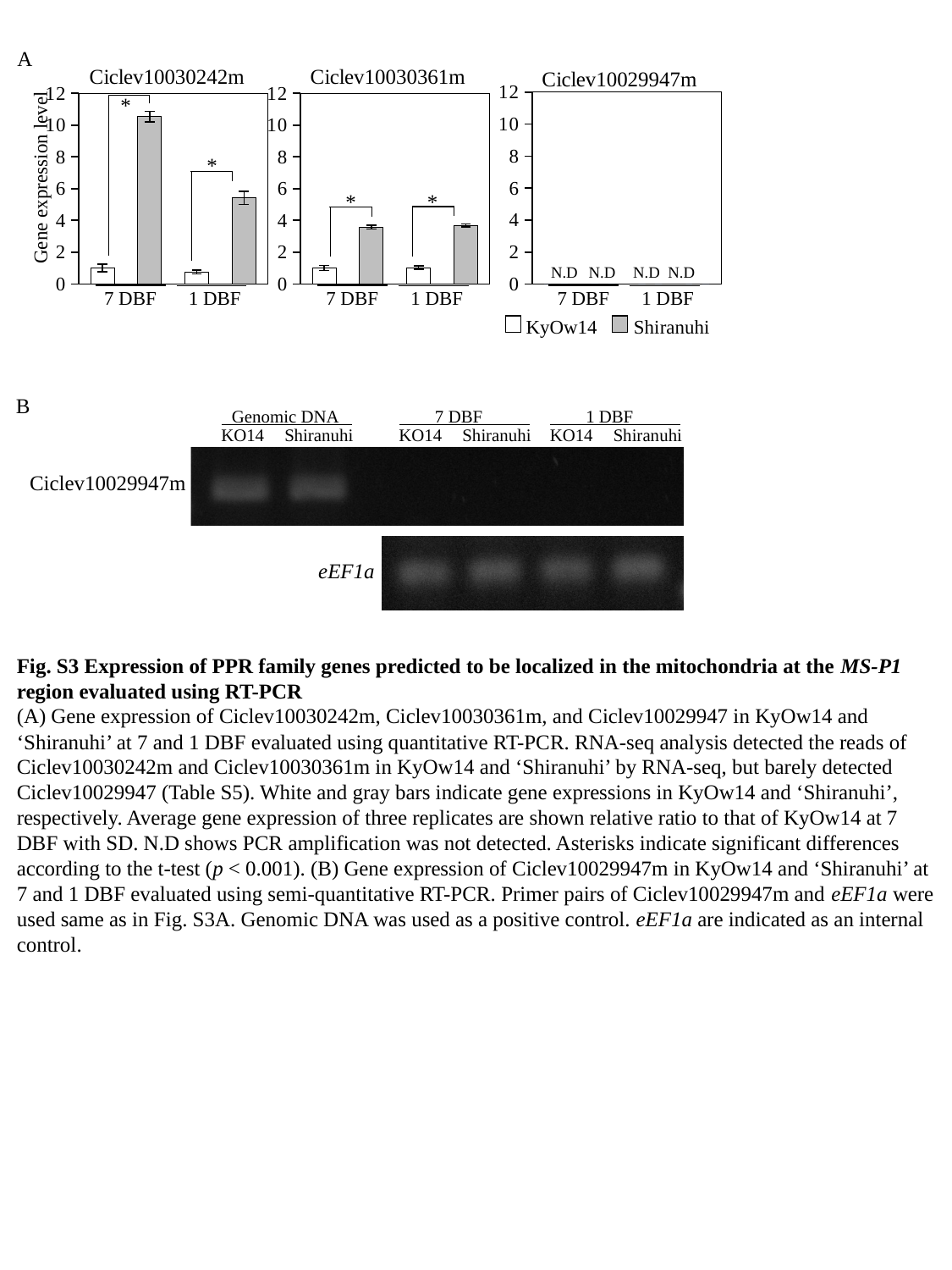

### Chart: Ciclev10029947m
| Category | |
|---|---|
| 7 DBF | 0.0 |
| 7 DBF | 0.0 |
| 1 DBF | 0.0 |
| 1 DBF | 0.0 |
### Chart: Ciclev10030242m
| Category | |
|---|---|
| 7 DBF | 1.0 |
| 7 DBF | 10.537859482868887 |
| 1 DBF | 0.7485066820611861 |
| 1 DBF | 5.413388256210801 |Gene expression level
7 DBF
1 DBF
7 DBF
1 DBF
KyOw14
Shiranuhi
### Chart: Ciclev10030361m
| Category | |
|---|---|
| 7 DBF | 1.0 |
| 7 DBF | 3.5677917340199428 |
| 1 DBF | 1.0206406622854571 |
| 1 DBF | 3.6738717013574553 |A
*
*
*
*
N.D
N.D
N.D
N.D
7 DBF
1 DBF
B
Genomic DNA
KO14
Shiranuhi
7 DBF
KO14
Shiranuhi
1 DBF
KO14
Shiranuhi
Ciclev10029947m
eEF1a
Fig. S3 Expression of PPR family genes predicted to be localized in the mitochondria at the MS-P1 region evaluated using RT-PCR
 Gene expression of Ciclev10030242m, Ciclev10030361m, and Ciclev10029947 in KyOw14 and ‘Shiranuhi’ at 7 and 1 DBF evaluated using quantitative RT-PCR. RNA-seq analysis detected the reads of Ciclev10030242m and Ciclev10030361m in KyOw14 and ‘Shiranuhi’ by RNA-seq, but barely detected Ciclev10029947 (Table S5). White and gray bars indicate gene expressions in KyOw14 and ‘Shiranuhi’, respectively. Average gene expression of three replicates are shown relative ratio to that of KyOw14 at 7 DBF with SD. N.D shows PCR amplification was not detected. Asterisks indicate significant differences according to the t-test (p < 0.001). (B) Gene expression of Ciclev10029947m in KyOw14 and ‘Shiranuhi’ at 7 and 1 DBF evaluated using semi-quantitative RT-PCR. Primer pairs of Ciclev10029947m and eEF1a were used same as in Fig. S3A. Genomic DNA was used as a positive control. eEF1a are indicated as an internal control.
