# Supplementary material for: Allelic haplotype combinations at the MS-P1 region, including P-class pentatricopeptide repeat family genes, influence wide phenotypic variation in pollen grain number through a cytoplasmic male sterility model in citrus
Source: Front Plant Sci. 2023 Jun 5;14:1163358. doi: 10.3389/fpls.2023.1163358 (PMC10278581; doi:10.3389/fpls.2023.1163358)
Supplement: Supplementary file 4 [file Table_1.docx]

|  |  | Number of seedlings | | | |  |
| --- | --- | --- | --- | --- | --- | --- |
| Cross combination | Grafting year | Population size | For evaluation of the number of pollen grains per anther | | |  |
|  |  |  | 2015 | 2016 | common | |
| Okitsu No. 46 × Kara (O46-K) | 2012 | 34 | 33§ | 28 | 28 | |
| Sweet spring × Okistu No. 56 (SS-O56) | 2012 | 31 | 30 | 28 | 27 | |
| Harehime × Okistu No. 63 (H-O63) | 2013 | 50 | 23 | 24 | 21 | |

**Table S1** F_1_ population used in this study.

The data marked with § were sourced from [1] and [2].

Reference

1. Goto S, Yoshioka T, Ohta S *et al*. Segregation and heritability of male sterility in populations derived from progeny of Satsuma mandarin. *PLoS One* 2016; **11**: e0162408.
2. Goto S, Yoshioka T, Ohta S *et al*. QTL mapping of male sterility and transmission pattern in progeny of Satsuma mandarin. *PLoS One* 2018; **13**: e0200844.
