# Supplementary material for: Allelic haplotype combinations at the MS-P1 region, including P-class pentatricopeptide repeat family genes, influence wide phenotypic variation in pollen grain number through a cytoplasmic male sterility model in citrus
Source: Front Plant Sci. 2023 Jun 5;14:1163358. doi: 10.3389/fpls.2023.1163358 (PMC10278581; doi:10.3389/fpls.2023.1163358)
Supplement: Supplementary file 7 [file Table_4.docx]

**Table S4** Summary of RNA-seq read number in the sterile selected strain, KyOw14, and fertile variety, ‘Shiranuhi’.

| Sample name | The number of toral reads | The number of  filtered reads (ratio) | The number of  mapped reads (ratio) |
| --- | --- | --- | --- |
| KyOw14 7DBF replicate 1 | 26,373,427 | 25,735,190 (97.58%) | 24,812,120 (94.08%) |
| KyOw14 7DBF replicate 2 | 25,369,888 | 24,547,904 (96.76%) | 24,004,988 (94.62%) |
| KyOw14 1DBF replicate 1 | 31,112,735 | 30,288,248 (97.35%) | 29,292,640 (94.15%) |
| KyOw14 1DBF replicate 2 | 21,848,808 | 21,095,024 (96.55%) | 20,640,569 (94.47%) |
| Shiranuhi 7DBF replicate 1 | 29,891,197 | 29,134,950 (97.47%) | 28,516,202 (95.4%) |
| Shiranuhi 7DBF replicate 2 | 21,431,041 | 20,719,530 (96.68%) | 20,458,071 (95.46%) |
| Shiranuhi 1DBF replicate 1 | 32,765,182 | 31,978,818 (97.60%) | 31,316,961 (95.58%) |
| Shiranuhi 1DBF replicate 2 | 20,047,802 | 19,354,148 (96.54%) | 19,135,627 (95.45%) |

7DBF: seven days before flowering, 1DBF: one day before flowering.
