# Supplementary material for: Allelic haplotype combinations at the MS-P1 region, including P-class pentatricopeptide repeat family genes, influence wide phenotypic variation in pollen grain number through a cytoplasmic male sterility model in citrus
Source: Front Plant Sci. 2023 Jun 5;14:1163358. doi: 10.3389/fpls.2023.1163358 (PMC10278581; doi:10.3389/fpls.2023.1163358)
Supplement: Supplementary file 8 [file Table_5.docx]

**Table S5** Normalized read counts in the pentatricopeptide repeat (PPR) family genes at the *MS-P1* region through RNA-seq.

|  | Normalized read counts | | | |
| --- | --- | --- | --- | --- |
|  | 7DBF | | 1DBF | |
| Accession No. | KyOw14 | Shiranuhi | KyOw14 | Shiranuhi |
| Ciclev10030242m | 40 | 247 | 47 | 157 |
| Ciclev10028481m | 49 | 138 | 58 | 73 |
| Ciclev10028233m | 35 | 77 | 37 | 47 |
| Ciclev10030082m | 101 | 400 | 156 | 181 |
| Ciclev10030279m | 66 | 94 | 54 | 54 |
| Ciclev10029914m | 37 | 199 | 40 | 116 |
| Ciclev10029947m | 0 | 5 | 0 | 0 |
| Ciclev10030361m | 65 | 272 | 83 | 208 |
| Ciclev10028181m | 72 | 159 | 77 | 93 |
| Ciclev10030145m | 3 | 7 | 1 | 0 |

7DBF: seven days before flowering, 1DBF: one day before flowering.
